# Breaking the Third Wall: Implementing 3D-Printing Techniques to Expand the Complexity and Abilities of Multi-Organ-on-a-Chip Devices

**DOI:** 10.3390/mi12060627

**Published:** 2021-05-28

**Authors:** Yoel Goldstein, Sarah Spitz, Keren Turjeman, Florian Selinger, Yechezkel Barenholz, Peter Ertl, Ofra Benny, Danny Bavli

**Affiliations:** 1 Institute for Drug Research, The School of Pharmacy, Faculty of Medicine, The Hebrew University of Jerusalem, Jerusalem 91120, Israel; Yoel.Goldstein@mail.huji.ac.il; 2 Faculty of Technical Chemistry, Institute of Applied Synthetic Chemistry, Vienna University of Technology, 1040 Vienna, Austria; sarah.spitz@tuwien.ac.at (S.S.); florian.selinger@tuwien.ac.at (F.S.); peter.ertl@tuwien.ac.at (P.E.); 3 Membrane and Liposome Research Lab, Hebrew University Hadassah Medical School, Jerusalem 91120, Israel; kerenbarhum@gmail.com (K.T.); chezyb1@gmail.com (Y.B.)

**Keywords:** 3D-printing, organ-on-a-chip, microfluidic, multi-organ-on-a-chip

## Abstract

The understanding that systemic context and tissue crosstalk are essential keys for bridging the gap between in vitro models and in vivo conditions led to a growing effort in the last decade to develop advanced multi-organ-on-a-chip devices. However, many of the proposed devices have failed to implement the means to allow for conditions tailored to each organ individually, a crucial aspect in cell functionality. Here, we present two 3D-print-based fabrication methods for a generic multi-organ-on-a-chip device: One with a PDMS microfluidic core unit and one based on 3D-printed units. The device was designed for culturing different tissues in separate compartments by integrating individual pairs of inlets and outlets, thus enabling tissue-specific perfusion rates that facilitate the generation of individual tissue-adapted perfusion profiles. The device allowed tissue crosstalk using microchannel configuration and permeable membranes used as barriers between individual cell culture compartments. Computational fluid dynamics (CFD) simulation confirmed the capability to generate significant differences in shear stress between the two individual culture compartments, each with a selective shear force. In addition, we provide preliminary findings that indicate the feasibility for biological compatibility for cell culture and long-term incubation in 3D-printed wells. Finally, we offer a cost-effective, accessible protocol enabling the design and fabrication of advanced multi-organ-on-a-chip devices.

## 1. Introduction

Microfluidic devices play an important role in numerous biological, chemical, and engineering applications [1,2,3,4,5]. One of those applications is the organ-on-a-chip technology (OOAC)—a discipline that focuses on the biomimetic emulation of tissue characteristics in a microfluidic device [6,7,8,9]. Due to its high potential, OOAC was selected as one of the “Top Ten Emerging Technologies” in the World Economic Forum [10]. Since OOACs enable the spatio- and temporal control over cellular microenvironments, they provide a more sophisticated platform for the cultivation of 3D-tissues compared with in vitro traditional cell culture set-ups. Advantages of OOACs include large surface areas, high mass transfer, low regent usage, fast mixing, rapid responses, high modularity, long-lasting viability, as well as precise control of physicochemical properties [1,8,11]. In recent years, increasing efforts have been directed towards multi-organ-on-a-chip platforms as the importance of systemic analysis approaches, including tissue-tissue interactions, has been off in the fields of drug screening [12] and personalized medicine [13], which require the interaction between different tissues to study the effects of a treatment on a whole system rather than focusing on a single tissue [13,14,15].

There is a vast range of methods for the fabrication of polymeric microfluidic based devices, such as photolithography, E-beam, laser cutting, and hot embossing, with each having distinguishing features useful for different applications [16]. Manufacture techniques can be classified into two main approaches: Templates for casting, and the fabrication of a whole device. To date, polydimethylsiloxane (PDMS) is the most widespread material for the fabrication of microfluidic devices thanks to its biocompatibility, elasticity, transparency, and low cost [15,17]. PDMS devices are generally derived by casting it on templates generated using traditional soft lithography methods [18,19]. Notwithstanding being common, soft lithography is a cumbersome method with multiple steps, that depends on the experience of the operator, is limited by its restricted height resolution, requires designated facilities, and is time-consuming. Recent advancements in the field of 3D-printing are bridging the gap to soft lithography which has long been considered the gold standard in microfabrication. Three-dimensional printing not only incorporates the benefits of the latter but in addition resolves its drawbacks (Table S1) [16]. Due to the constant evolution of the technology it has the potential to significantly change the way we design and manufacture microfluidic devices [20,21].

**Figure 1:**
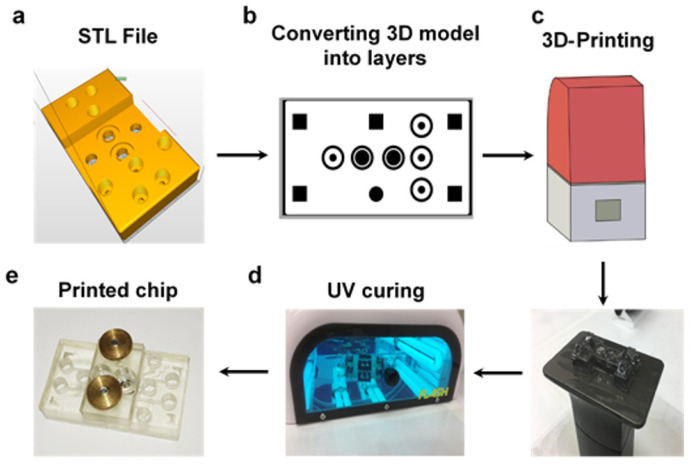
3D-printing process. (**a**) STL file was imported to Asiga’s composure (**b**) the model was sliced into layers with a fixed height. (**c**) During the printing process, each layer was separately exposed to UV light at a predetermined exposure time. (**d**) The 3D-printed object was rinsed with isopropanol and fully cured in an UV oven to get the (**e**) final product.

Digital light processing (DLP) 3D-printing enables the fabrication of multilayered and maskless-based microfluidic structures, at a high resolution, when its printed products are characterized with high precision [22,23]. This method uses an array of micromirrors to transmit UV light from a light projector to perform selective curing of a polymer resin and thereby convert it to the required geometry. Prior to printing, the CAD design is converted to a STL file and then sliced to multiple layers at a fixed thickness which are converted to black and white masks (Figure 1). In accordance with soft lithography, during projection, the white pixels in each mask activate corresponding micromirrors to transmit light that triggers the curing of the resin, while the black pixels do not transmit light and therefore do not activate curing [24]. Post-printing the printed object requires further rinsing and curing in order to complete the process (Figure 1). In total, the fabrication process from an initial concept to a 3D-printed product takes a relatively short period of time, ranging from a couple of hours to a couple of days. Having said that, the DLP resolution is limited to its pixel size, and therefore, in terms of channel and feature resolution, it is currently inferior to photolithography and 3D micro- and nanostructuring methods, such as two-photon polymerization [25,26,27]. Since our proposed designs’ channels and features ranged between 100 µm and several mm, this disadvantage is insignificant. 

Here, we present a multifunctional, durable, and easy to use microfluidic device as a OOAC platform. We used an Asiga DLP MAX X 3D printer to fabricate a generic multi-organ-a-chip (MOC) device in two methods, one that contained a PDMS based microfluidic unit and one with embedded 3D-printed microchannels. Both approaches enable culturing and supporting three different cell types in three different compartments, while facilitating the interaction between each of the individual compartments through interconnective microfluidic channels. This methodology allowed us to achieve a complex architecture that was nearly impossible to produce and maintain using soft lithography. Moreover, our findings suggest the possibility of using commercial 3D-printing resin (DETAX Luxaprint) as a biocompatible platform for cell culture. Finally, this study introduces an accessible and easy to use method for setting up platforms to study the interaction between multiple organs such as the gut-brain axis in Parkinson’s disease and tumor-on-chip, while enabling the flexibility to treat each organ with its own nutrient requirements and shear stress, potentially bringing us one step closer to a high functional in vitro model for biological systems.

## 2. Materials and Methods

### 2.1. 3D-Printing

#### 2.1.1. 3D-Printer and Software

The microfluidic device was fabricated with a digital light processing stereolithography printer (Asiga Max-X27 UV, Sydney, Australia). This 3D-printer has a LED light source with 385 nm UV wavelength. The XY pixel resolution of the printer’s projector is 27 μm with a minimum Z plane resolution of 1 μm. The maximum build footprint is 51.8 × 29.2 × 75 mm (X, Y, and Z). All the objects were designed in Autodesk AutoCad^®^ (Q.70.0.0 AutoCAD 2020, San Rafael, CA, USA). The final design was exported as a stereolithography (STL) file and uploaded to the printer’s software: Asiga composer for 3D- printing (version 1.1.7, 2020, Sydney, Australia). The device was printed with the Detax Luxaprint^®^ Mould Clear resin (wavelength 385 nm).

#### 2.1.2. 3D-Printing Procedure

Prior to printing, the vat was filled with resin and positioned under the build platform. The printing process is then carried out as follows: First, the platform surface lowers into the vat to a predetermined height and the DLP projects the first layer of the design for a predetermined time. The platform then elevates above the surface of the vats for a few seconds before dipping again into the vat for the formation of the next layer based on the CAD design. This sequence is repeated until the entire design is printed. The printed object is removed from the platform, the uncured resin is removed by washing the model with isopropyl for 3 min in a bath sonicator (Bandelin, Germany), dried using air pressure, and cured in an UV oven for 5 min (PCU Led, Dreve, Germany).

### 2.2. MOC Device Fabrication

#### 2.2.1. PDMS-Based Microfluidic Unit Fabrication Using 3D-Templates

**Figure 2:**
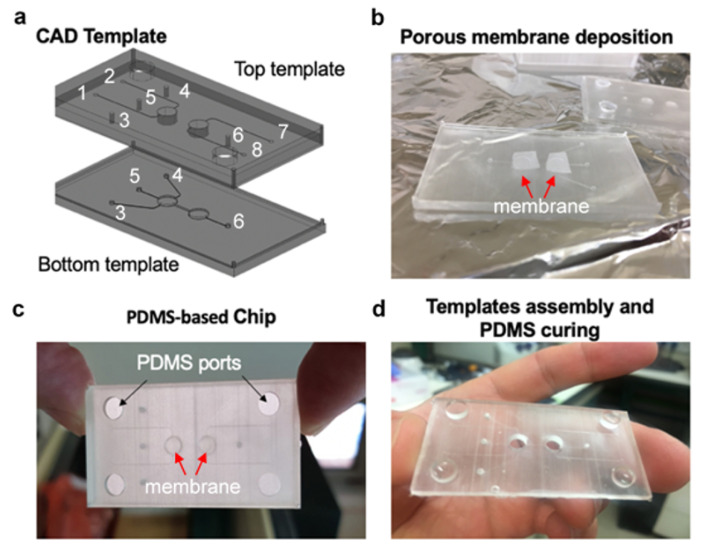
PDMS-based MOC’s disposable unit fabrication. (**a**) A set of two compatible templates was 3D-printed, containing 4 coupled inlets and outlets: {1,2}, {3,4}, {5,6} and {7,8}. While ports {5,6} connect the cell culture compartments, the remaining ports enable controlled perfusion of fluid to each separate compartments, when ports {1,2}, {7,8} perfuse the two upper wells and ports {3,4} perfuse the lower well. (**b**) Before assembled two pieces of porous membrane (red arrows) were located on top of the wells’ template, so when connected (**c**) the pieces were locked between them (red arrows). PDMS was then poured into the hollow chamber through 4 ports and underwent overnight curing at 65 °C. (**d**) After peeling, the PDMS was punched and Plasma-bonded to a glass slide at its bottom side.

Templates for casting PDMS (Sylgard^®^ 184, Dow corning, Midland, MI, USA) based chips were 3D-printed and included a top and bottom unit, that contained within them their own wells (pore size 3.85 mm) and channels (0.1 × 0.1 mm) (Figure 2). Following template printing, a porous membrane (It4ip, Belgium; PET, thickness: 23 µm, pore size 0.45 µm, pore density 2 × 10^6^/cm^2^) was placed and locked between the top and bottom units’ printed microwells to allow separation between the upper and lower wells. Following membrane deposition, the upper template unit was closed above the bottom template. The PDMS at ratio 1:10 was inserted via the PDMS ports and left to cure at 65 °C overnight. Once the PDMS was cured, it was covalently bonded to top and bottom glass slides using Femto oxygen plasma activation (Diener, Germany) for 15 s at 50 W.

#### 2.2.2. Whole 3D-Printed Device

A set of a top and bottom unit, which was 3D-printed as described above (Section 2.1.2), contained wells (pore size 3.85 mm) and channels (1 mm radius). Following the printing procedure, a small amount of resin was gently applied on top of both units and a porous membrane was placed above the wells of the bottom unit. The two units were combined and secured with metal clips and the resin was cured in an UV oven for 5 min.

### 2.3. Computational Fluid Dynamics (CFD) Simulation

CFD simulations were performed using the simulation software Autodesk CFD (2021). Prior to the simulation, the geometry of the microfluidic device was split into 4.4 × 10^6^ control volumes using the automatic mesh generator. All wall boundaries were assumed as ideally smooth, and no slip boundary conditions (zero flow velocity at the wall) were selected for all surfaces. Outlet pressures were set at atmospheric pressure p = 1 bar (100 kPa). The free area ratio of the integrated membranes (PET) was calculated based on the manufacturer’s design parameters employing the following formula: f = AopenAtotal. Simulations were performed assuming isothermal flow as well as Newtonian fluid behavior, including constant dynamic viscosity and constant density (incompressible). As the concentrations of the dissolved species in the fluid are low, the properties of the solvent and water have been used for the simulation (ρ = 993 kg/m^3^, η = 0.001003 Pa·s at 37 °C).

### 2.4. Cell Culture

PC9 GFP lung adenocarcinoma cells were kindly provided by Dr. Oren Ram (The Hebrew University, Jerusalem, Israel). The cells were grown in DMEM (Sigma-Aldrich, Steinheim, Germany, cat. no. D5671), supplemented with 10% fetal bovine serum (FBS; Biological Industries, Beit Haemek, Israel, cat. no. 04-007-1A), 50 μg/mL penicillin-streptomycin (Biological Industries, Beit Haemek, Israel, cat. no. 03-031-1B), 2 mM L-glutamine (Biological Industries Israel, cat. no. 03-020-1B), and 1 mM sodium pyruvate (Biological Industries Israel, cat. no. 03-042-1B), and were incubated at 37 °C in a humidified incubator with 5% CO_2_.

### 2.5. Cell Viability and Biocompatibility Evaluation

Prior to cell seeding, the 3D-printed device was sterilized with 70% EtOH and 30 min exposure to UV light. The biocompatibility evaluation was performed with a multicellular spheroids model. After cell detachment, 6000 cells were seeded into agarose wells (20 mg agarose dissolved in 1 mL 0.9% NaCl), fabricated in micro-mold petri dishes (MicroTissues 3D Petri Dish micro-mold spheroids, size s, Merck, Germany) and in hanging drop cultures within the MOC. The spheroids were cultivated for 72 h without perfusion by placing the device in a humidified incubator at 37 °C with 5% CO_2_. The cell viability was monitored with a 24 h interval by measuring the GFP signal within the cells to compare the standard technique with the on chip formation.

## 3. Results

### 3.1. MOCs Design Guidelines

In this study, we have designed and fabricated a microfluidic multi-organ-on-a-chip device that can incorporate multiple cellular cultures, including 3D cellular aggregates, such as spheroids and/or cell monolayers under individually controlled flow conditions (Figure S1). The microfluidic device was designed in such a manner that it should include two sets of upper and bottom wells, separated with an optically clear PET-based porous filter (on which the cells are seeded), interconnected by a microchannel in a transparent housing with a bottom glass layer. Optical considerations dictated that the distance between the porous membrane and the bottom of the device should be 1 mm, to allow for both optical and fluorescence microscopy, using long-distance objectives. A major engineering consideration was directed towards the fabrication of a device that allows for the procedures of cell seeding and handling to be as simple as possible. In such a design, the cells/spheroids/organoids are seeded within the separate wells either in an open configuration or by internal seeding using microfluidic channels. The open configuration of the MOC simplifies the introduction of cells and spheroids into the device. Moreover, the well’s dimensions and the separation of the compartments by the PET membrane make it very challenging to use common spheroid’s self-assembling designs [28,29]. In addition, the open configuration provides an important advantage of accessibility to the samples, in order to perform various analysis, e.g., genomics and RNA seq, that are inaccessible using close designs. Another requirement was that each well has its own inflow/outflow, which allows the ability to adjust the flow rates and nutrients needed for each cell type, while protecting the cells from the shear flow inside the well. The last necessity was to design a configuration that would allow controlling and manipulating the interaction between the different cell compartments.

### 3.2. PDMS Based MOCs Fabrication

PDMS-based devices are commonly used for microfluidic applications. PDMS is biocompatible, optically clear, and gas-permeable [15,17,30]. Traditionally, PDMS-based devices are casted on molds that are fabricated by photolithography of SU-8 on silicon wafers. Despite its advantages, this method is limited to a 2D or semi-2D structural design, and therefore not suitable to our requirements. To overcome it, and to simplify the template fabrication, we first evaluated the ability to fabricate the MOC with a PDMS based unit that was casted on 3D-printed templates. Separating two parallel microchannels by the means of integrating a porous substrate has been successfully shown to enable the analysis of critical physiological parameters such as tissue barrier function, transcellular transport, absorption, and secretion in a variety of OOAC applications including devices for the lung [31], the intestine [32], the blood-brain-barrier [33], as well as for microfluidic metastatic invasion assays [34]. Therefore, we 3D-printed an upper and a bottom template to allow for the integration of any type of porous membrane within the device (Figure 2a–c). While the upper template included two integrated columns (which were translated into wells at the PDMS replica) and two coupled sets of inlets and outlets (one set per well (Figure 2a)), the bottom template contained two integrated columns as well as two sets of coupled inlets and outlets, one set for cell seeding only and a second set for fluidically connecting the two individual wells (Figure 2a). The two templates were designed to complement one another so that when aligned they created a hollow chamber for liquid PDMS insertion (Figure 2b). This method facilitated the assimilation of the membrane by capturing it between the upper and bottom columns, locking it in place while the PDMS polymerized around it during curing (Figure 2c,d). After curing, a glass slide was attached to the bottom side of the PDMS employing plasma bonding. To further facilitate the use of the PDMS-based device, we 3D-printed a complementary reusable housing platform. It included an upper cover that contained eight embedded screws compatible with flangeless nuts aligned with the PDMS inlets and outlets, two glass-based viewing windows aligned with the wells, and a bottom cover with a large viewing window (Figure S2). In addition, to decrease leaking between the units we designed hollow crevices around the pores at the inner side of the upper cover that were tailored to O-rings (Figure S2).

### 3.3. 3D-Printing MOCs Fabrication

**Figure 3:**
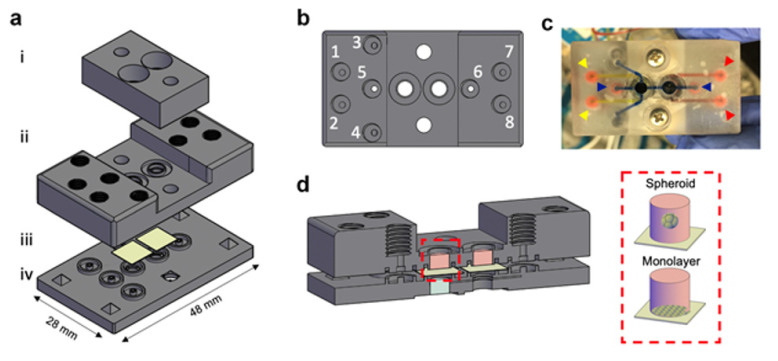
Whole 3D-printed based MOC design. The aim of the MOC is to facilitate the culture of different cell types in different compartments which are connected by a micro-channel while enabling separate controlled perfusion. (**a**) Disassembled illustration of the MOC platform, visualizing the three 3D printed parts (i, ii, iv) and the membrane (iii). (**b**) The device has 4 coupled inlets and outlets: {1,2}, {3,4}, {5,6} and {7,8}. While ports {5,6} connect the cell culture compartments, the remaining ports enable controlled perfusion of fluid to each separate compartment. (**c**) Perfusion of three colors (yellow, red and blue arrowheads) through ports {1,2}, {5,6} and {7,8} (ports {3,4} are blocked) demonstrates unmixed flow. (**d**) Cross section visualization of the MOC, presenting the three compartments for cell seeding, a lower well (light blue) and two upper wells (pink). The upper wells (red rectangle) are design in an open configuration, enabling direct seeding of monolayer or of a spheroid/organoid.

Following the fabrication of the 3D-printed molds for deriving the PDMS devices, we evaluated the ability to fabricate a completely 3D-printed device of similar design including an integrated perfusion system (Figure 3a,b). The chip was designed as two individual units so that a porous membrane can be placed between them, separating the integrated wells into four compartments with separate perfusion options for each of the individual chambers (Figure 3c). The two units were then bonded using a liquid state resin that served as an adhesive following UV exposure. Two 0.15 mm glass slides were glued the same way to the bottom part of the lower wells, sealing them. In order to seal the upper wells, a cover unit was 3D-printed, which contained two glass-windows aligned with the wells. Similar to the PDMS-based MOC design, here also, in order to decrease leaking, O-rings were inserted to both the upper side of the chip’s wells and the internal side of the cover’s windows.

In both MOCs configurations, the upper wells enabled open access for cell seeding, while the lower well enabled cell seeding only by microfluidic channels (Figure 3d). As mentioned, the MOC has four sets of paired inlets and outlets (Figure 2a and Figure 3b). For operating the MOC, cells must be seeded first as a monolayer on the underside of the porous membrane. To do this, the MOC should be inverted while cells are inserted into the chip through ports 3 and 4 with ports 5 and 6 blocked, followed by overnight incubation to enable the cells to sink and attach to the membrane. Once the cells have attached to the membrane, the MOC can be turned over and cells can be seeded in the two upper wells. After seeding both compartments of the device the MOC is sealed using screws (Figure 1e and Figure S3).

This methodology allows for the seeding of distinct cell types in the different compartments without unwanted mixing of the individual cell types. In order to connect the three, ports 3 and 4 are blocked, while ports 5 and 6 are used to insert the medium into the lower wells, creating a conjoint flow (Figure 3b and Figure S3).

### 3.4. Shear Force Protection

Depending on their place of origin in vivo, cell types are exposed to a variety of different biophysical stimuli and microenvironments which strongly impact the cell’s/tissue’s intrinsic physiology. For example, the hepatocyte function is damaged at a shear stress > 5 dyne/cm^2^, while oxygen uptake rates can reach 0.9 nmol/s per 10^6^ cells [35]. Additionally, prior work demonstrated that endothelial cells change their morphology and gene expression based on the shear flow. Small capillary systems need shear flows of ~10 dyne/cm^2^ for normal gene expression [36], while intestinal [37] and neuronal [38] function is damaged at a shear stress > 5 dyne/cm^2^. Aside from shear stress requirements, cells also differ in their metabolic and oxygen requirements. For example, oxygen uptake rates can reach up to 0.2 nmol/s per 10^6^ endothelial cells [39], 0.88 nmol/s per 10^6^ neuronal cells [40], and 1.2 nmol/s per 10^6^ intestinal cells [41]. In order to account for these differences in oxygen and nutrient demand flow rates need to be tailored to each individual cell type. To that end, we designed the MOC in a way that each “cell type” has its own inflow/outflow allowing the adjustment of oxygen, nutrient, and shear force as needed without harming any of the other cell types. Inter-tissue cross-talk within the microfluidic device is enabled by connecting the individual membrane-separated compartments with a connective microchannel.

**Figure 4:**
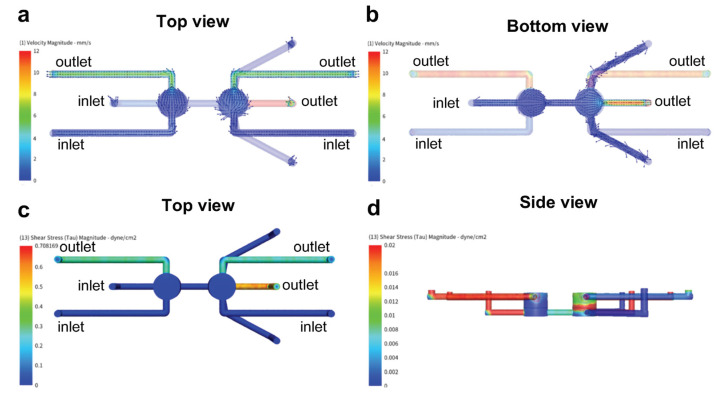
(**a**) Flow velocity profile of chamber 1 and chamber 2 at a perfusion rate of 10 mm^3^/min or 1 mm^3^/min, respectively. (**b**) Flow velocity profile of the connective channel at a perfusion rate of 10 mm^3^/min. (**c**) Overview of the shear stress profile generated within the microfluidic device. (**d**) Overview of the shear stress distribution along the vertical axis of chamber 1 and chamber 2.

To analyze the fluid behavior (e.g., shear stress, flow velocity) within the designed microfluidic device a CFD simulation was performed. To that end, a volume flow rate of 10 mm^3^/min was applied in the first well compartment (chamber 1) and the connective microfluidic channel, while the volume flow rate within the second well compartment (chamber 2) was set at 1 mm^3^/min. As depicted in Figure 4a,b, a laminar flow profile with an average flow velocity of 0.72 mm/s could be established in both top compartments as well as in the bottom connective channel. Overall, the flow velocity can be divided as follows: 77% of the volume of the microfluidic device experience a flow velocity below 0.62 mm/s, 13% a flow velocity between 0.62 and 2.48 mm/s, while the remaining 10% are exposed to a flow velocity in the range of 3.08 to 12.34 mm/s. Peaks in flow velocity are observed at the outlet of each of the three individual microfluidic compartments. Similarly, elevated shear stresses are located at the outlets of the device (Figure 4c), with the highest shear stress of about 0.65 dyne/cm^2^ obtained within the last third of the connective bottom channel. The comparatively big geometrical features (Ø 4 mm, h: 3 mm) of the two cell culture compartments result in low shear stresses throughout the center of the cylindrical chamber. In chamber 1, the average shear stress equals 0.002 dyne/cm^2^, while chamber 2 displays an average of 0.01 dyne/cm^2^ (Figure 4d). It has to be noted that the establishment of low shear stresses is an essential prerequisite for the cultivation of sensitive cellular constructs, such as brain organoids that are sensitive to high mechanical stresses. On the other hand, the differentiation and maturation of cellular barriers, such as the gut and the blood-brain-barrier, require physiological shear stresses for maturation and proper functionality. To that end, the shear stresses located in close proximity to the integrated membranes were further examined (Figure S4). While the maximum shear stress on the surface of membrane in chamber 1 is situated at about 0.001 dyne/cm^2^ with lower shear stresses (0.0001−0.0003 dyne/cm^2^) at the border of the membrane, the maximum shear stress obtained within the surface of chamber 2 compartment is 20× fold higher with a shear stress of 0.02 dyne/cm^2^. The ability to generate significant differences in shear stress exposure between the individual cell culture compartments provides an optimal precondition for the development of several organ models, each with a desirable shear force.

### 3.5. 3D-Printed Based MOC Evaluation and Cytotoxic Effect of (Meth)Acrylate Resin

**Figure 5:**
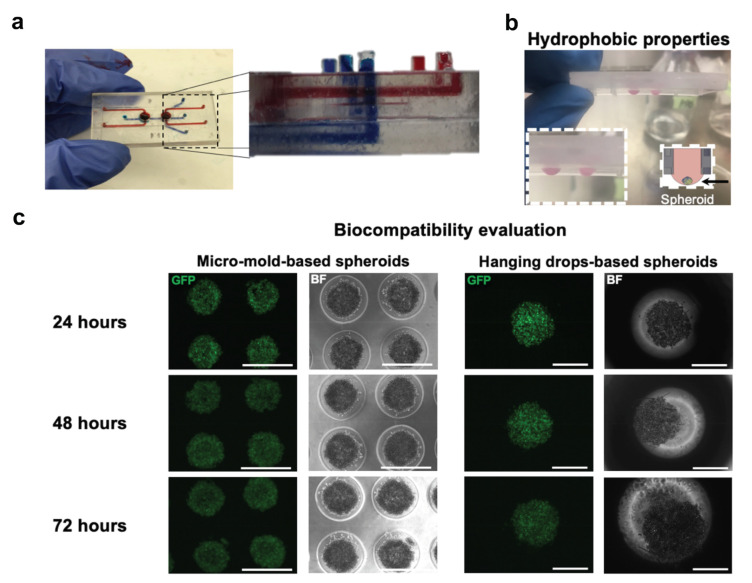
MOC evaluation. (**a**) Disposable unit composed of 3D-printed top and bottom layers bonded using UV-based biocompatible (meth)acrylate resin. Red (top layer) and blue (bottom layer) colors stayed separated over a week which confirms the bonding efficiency. (**b**) The hydrophobic surface properties of the device result in a high contact angle of aqueous liquids, allowing the formation of spheroids using hanging drop cultures. (**c**) GFP-PC-9 cells confirmed the formation of spheroids within the device and was compared to spheroid formation in micro-mold-based methodology. Cell viability evaluated over 3 days (72 h) suggesting on the biocompatibility of the UV-based (meth)acrylate resin used. Scale bar: 500 µm and 300 µm for micro-mold-based spheroids and hanging drop-based spheroids respectively.

To confirm that there is no leakage between the layers after bonding, red (top layer) and blue (bottom layer) colored water was perfused through the system for a duration of 1 week, revealing no leaks throughout the entire system (Figure 5a). Due to the hydrophobic surface properties of the (meth)acrylate resin, aqueous liquids adhere to the device allowing for the use of the hanging drop methodology and self-assembled formation of spheroid/organoids within the device (Figure 5b and Figure S5). Although spheroids can be introduced to the MOC externally and manually through the open wells, the possibility of cell assembly on the chip confers advantages, such as simplifying the process and eliminating destructive steps of spheroid handling.

The DETAX Luxaprint 3D clear mould resin was FDA approved for medical Class IIa devices and is widely used for the fabrication of hearing devices. Hence, it was essential to test whether it is biocompatible also for microfluidic/organ-on-a-chip applications. As a preliminary assay for testing the resin biocompatibility, the viability of GFP-PC9 cells was tested for a period of 3 days by means of fluorescence intensity (Figure S6). When these GFP labelled cells die, they lose their ability to synthesize the GFP protein and therefore cannot be seen using standard fluorescence microscopy. We compared a standard technique for spheroids formation using agarose wells with the self-assembled formation protocol for spheroids using hanging drops within 3D-printed wells. After seeding, cells were incubated for 24 h to allow for the formation of spheroids in hanging drops, prior to the incubation for an additional 3 days. Our results revealed no significant loss in GFP intensity for cells incubated in agarose wells compared to spheroids generated and incubated within the 3D-printed wells (Figure 5c), suggesting that the resin seems to be a good candidate for microfluidic/organ on chip applications.

## 4. Discussion

The understanding that animal models and traditional 2D cell culture often fail to mimic human physiology, together with the growing pressure to minimize animal experiments, emphasize the need for better platforms to emulate in vivo conditions [42,43]. OOAC microfluidic devices have the potential to meet this need, which is why the field has been extensively growing over the last decade. OOAC can be categorized into two concepts, single-organ-on-a-chip devices and multi-organ-on-a-chip devices. While single-organ-on-a-chip devices are designed to study a specific tissue, multi-organ-on-a-chip devices are designed to integrate several tissues or cell lines in one connected platform to enable a more accurate model by allowing for crucial physiological criteria such as reciprocal signaling [13,15,43,44,45].

Single-organ-on-a-chip models have been proposed for almost all organs, such as the gut [32], liver [46,47], lung [31], heart [48,49], and brain [50]. Each device is tailored to create an optimized platform for each individual specification and requirement attributed to the desired tissue. Although those designs enabled a significant improvement in mimicking in vivo conditions, by focusing on a specific tissue or cell line they lack a systemic context including cross organ communications, which are crucial to many applications such as pharmacokinetics and drugs screening, for example [12]. Multi-organ-on-a-chip devices, on the other end, are designed to tackle this issue by interconnecting different tissues with one another. One of the challenges of such a device is to maintain the balance between the combination of multiple tissues or cell lines, respectively and the need to provide an optimal growth environment for each component. The two foremost approaches for designing multi-organ-on-a-chip devices include (i) the coupling of two or more single-organ-on-a-chip devices using capillary connections [15] and (ii) the integration of multiple tissues into one platform. The medium in these chips is usually perfused in one direction employing a common fluid stream or in a closed-circuit system that enables media recirculation, which mimics reciprocal interactions. As noted, different cells and tissues have different requirements such as specific nutrients (hormones, vitamins, and growth factor), external stimuli and oxygen consumption [35,51,52,53]. Therefore, multi-organ-on-a-chip devices that use perfusion of media either in a one way or recirculating common flow can have a problem to meet these requirements. The ability to create different physiological environments for each organ under conditions of joint flow, is very challenging and demands delicate mechanical manipulations of the micro-channels’ dimensions, as well as manipulations on the medium itself. More advanced designs use an approach in which the different cells or tissues are separated in different compartments and manipulate medium flow by gravity [12] or by complex pump configurations [54].

A major consideration that guided us when designing the MOC was to enable an independent control over the conditions of each compartment, without affecting other compartments. To do so, we designed the MOC to have separate inlets and outlets of every functional compartment. Moreover, we used porous membranes to create a barrier within the chip and to create two sets of coupled compartments. Incorporating such membranes in microfluidic devices allows cells to be seeded (Figure S6) alongside fluid passage [55]. Our concept was reinforced by computational fluid dynamic simulation (Figure 4) showing that flow can be separately manipulated through each inlet without affecting both the velocity and shear stress of the other compartments. Hence, it showed that not only can we control what medium every compartment will be perfused with, but also the external stimuli (shear stress) that is needed for optimal conditions.

Here, we presented two optional methods for fabrication of the MOC, one with a PDMS based microfluidic core and the second with 3D-printed embedded microfluidics channels, each with its own advantages and disadvantages. While PDMS is biocompatible for cell culture, there is no available data regarding commercial 3D-printing resins cytotoxicity (despite some of them being classified as biocompatible). Indeed, it was shown that different photopolymers for 3D-printing have different cytotoxic effects on cells, ranging from very low to high [56]. Applications such as the MOC in which cells are exposed to culture medium for days, are more exposed to leachable cytotoxic effects than devices that are used for short-term applications. Hence, it is necessary to evaluate the cytotoxicity of the resin on cells, despite being classified as medical device Class IIa. Here, we showed that GFP-PC9 cells seemed to keep their GFP intensity, displaying similar viability compared to cells incubated in agarose wells (Figure 5). However, these findings are preliminary, indicating the potential biocompatibility of the resin. Additional experiments are needed in order to evaluate the long-term effect of the resin on cell viability, functionality, and gene expression. On the other hand, PDMS is a porous hydrophobic material, which means that it can absorb small molecules [30,57,58] as opposed to 3D-printed objects that cannot. There are other limitations worth noting in each of the two methods, such as potential defects in PDMS demolding (of either photolithography-based or 3D-printing-based molds) when using the casting technique, and as a limited minimum channel size (couple of hundreds of microns) when 3D-printing whole devices, using DLP [22,59]. In view of this, it is necessary to select the method of fabrication suitable for the purpose of the specific experiment. For example, the use of PDMS-based devices for long-term drug screening or for pharmacokinetic purposes, can interfere with the experiment outcome, making it unfitting for such applications.

The progress in the field of 3D-printing enables the development of new and creative approaches that were impossible using traditional photolithography or that acquired special, complicated, and potentially costly methods. We utilized the advantages of 3D-printing to prototype and remodel our designs, in a relatively short period of time, to converge on a design that will be as comfortable as possible for the user. The offered devices are designed as bioreactors that were aimed to study the interaction between two or three cell lines and tissue combinations, enabling the culturing of cells using microfluidic perfusing in the lower compartment and an open seeding configuration in the upper ones. When assembled, the permeable membranes and the lower collecting channel enable the interactions between the different compartments. Currently, systemic context and cross organ communications were already achieved and shown by many other multi-organ-on-a-chip designs. However, thanks to 3D-printing, our MOC design not only allows that, but also provides the tools for better mimicking in vivo conditions, by enabling independent control over the conditions of each cell compartment. In addition, follow up studies will be conducted to extend our study to different and well-defined organs, together with a more in-depth examination of 3D-resin cytotoxicity for this purpose. The current designs can be easily replaced, and fabricated in a matter of hours, allowing great flexibility and creativity to adjust the right platform to the right needs. This work emphasized the importance of 3D-printing as a simple and affordable prototyping tool for the development of advanced microfluidic and culture devices.

## Data Availability

Not applicable.

## Supplementary Materials

The following are available online at https://www.mdpi.com/article/10.3390/mi12060627/s1, Figure S1: Explosive view of a theoretical multi-organ microfluidic device, Figure S2: Illustration of the microfluidic platform, Figure S3: A top view of the assembled whole 3D-printed MOC, Figure S4: Shear stress simulation on the membrane surface, Figure S5: 3D-printed unit used to evaluate cell biocompatibility and toxicity, Figure S6: Fluoresce Visualization of viable GFP-PC9 cells, Table S1: 3D printing characteristics compared with common microfluidic based methods.
