# Supplementary material for: Breaking the Third Wall: Implementing 3D-Printing Techniques to Expand the Complexity and Abilities of Multi-Organ-on-a-Chip Devices
Source: Micromachines (Basel). 2021 May 28;12(6):627. doi: 10.3390/mi12060627 (PMC8227399; doi:10.3390/mi12060627)
Supplement: Supplementary file 1 [file micromachines-12-00627-s001.zip › micromachines-1222719-supplementary.pdf]

Supplementary Information for “Breaking the 3rd wall: implementing 3D printing technics to expand the complexity and abilities of multi-organ-on-a-chip devices“

Table S1. 3D printing characteristics compared with common microfluidic based methods

Method	Approach	Polymer	transparency	Biocompatible	Permeable	Architecture	Cost
**Soft lithography**	Template	PDMS	High	Yes	Yes	2D	Moderate
**E-beam**	Template	PDMS	High	Yes	Yes	2D	High
**Hot embossing**	Template	PMMA<br>PC<br>COC	High	Yes	No	Semi-2D	High
**DLP – 3D printing**	Whole device	PEGDA	moderate	Yes	Yes	3D	Low
	Template/ whole device	Detax Luxaprint Mould Clear	moderate	NA	No	3D	Low
**Laser**	Whole device	Glass	High	Yes	No	3D	High

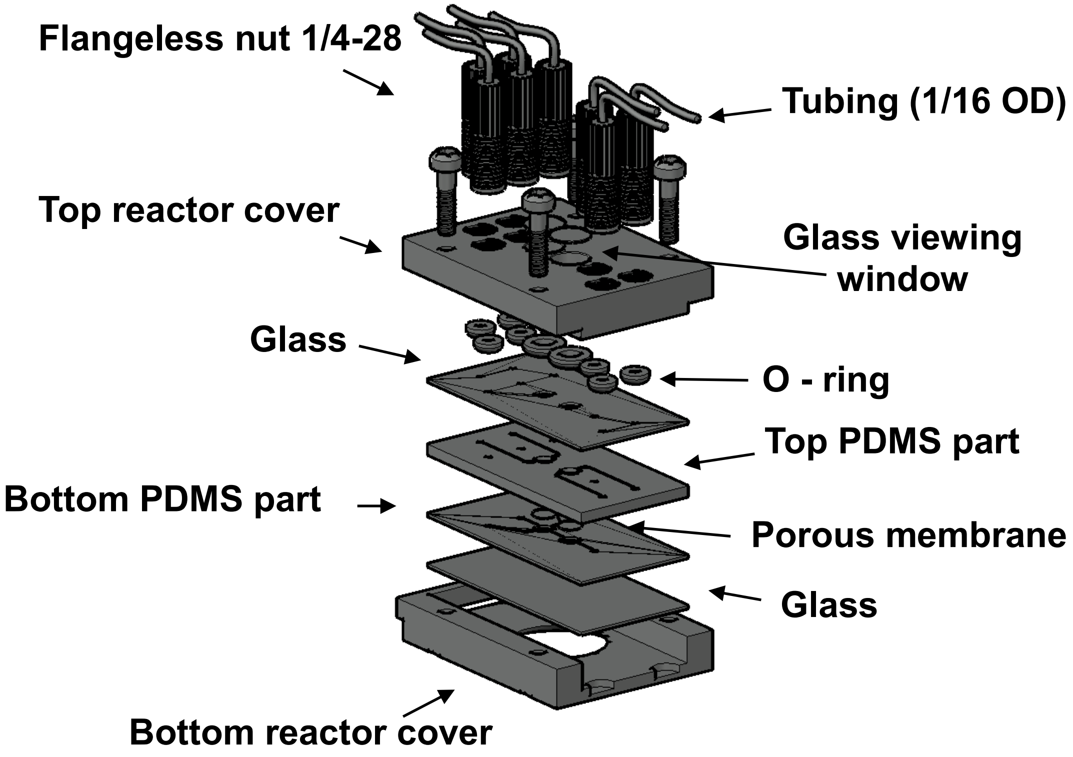

Fig. S.1. Explosive view of a theoretical multi-organ microfluidic device.

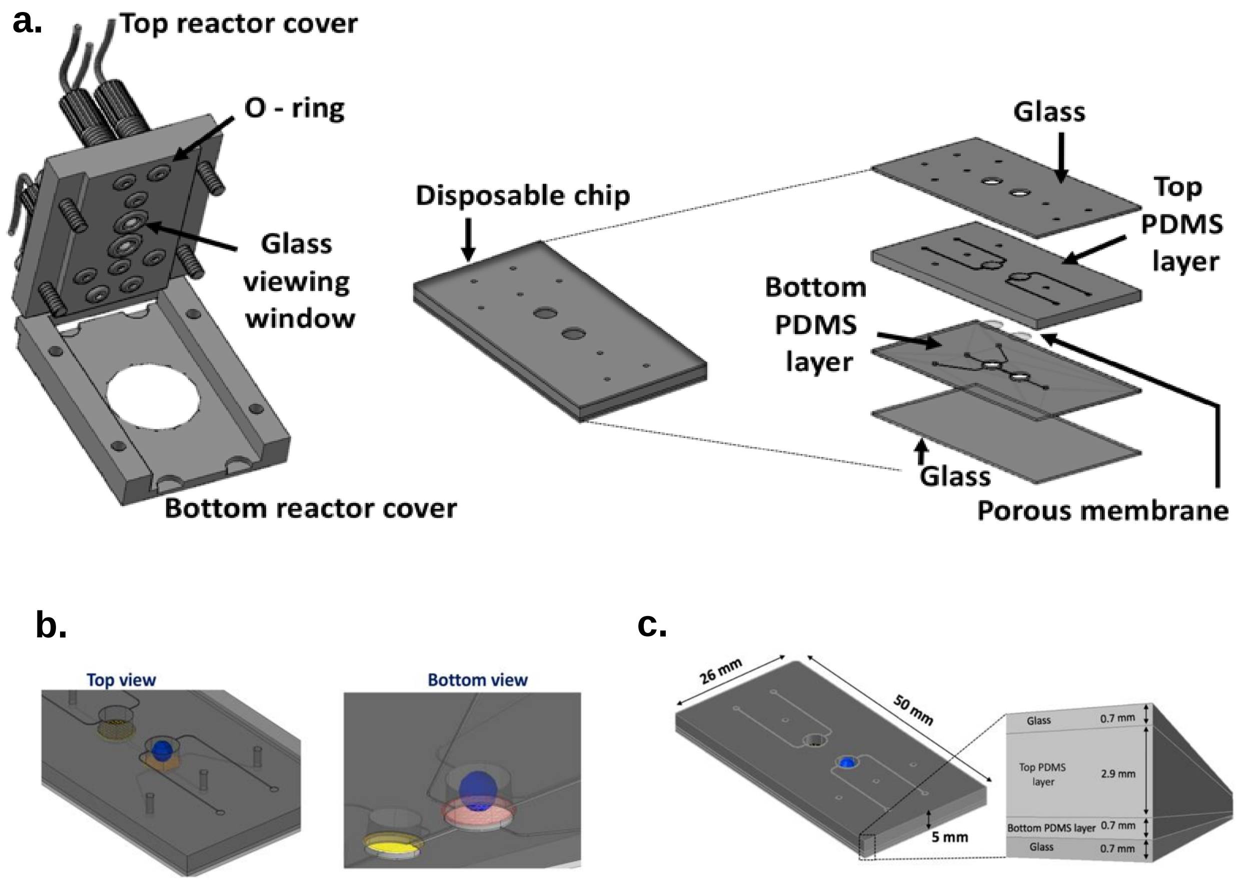

Fig. S.2. **Illustration of the microfluidic platform**. **a.** The chip is composed of a fixed (external) and disposable (internal) units.In such a design the fixed unit is 3D printed while the disposable unit is made of PDMS-casted on 3D printed templates. **b.** The system supports cell seeding (monolayer/organoids) in an open configuration within the disposable unit and perfusion via the external unit. Each well has its own inflow/outflow while interaction between the wells is done via the bottom connecting channel. **c.** Illustration of the disposable unit layers and dimensions.

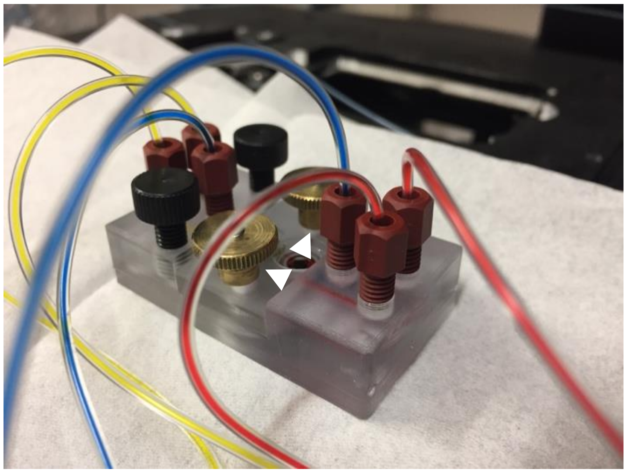

Fig. S.3. A top view of the assembled whole 3D-printed MOC. The assembled of the MOC was done by using screws (white arrowheads). Red, blue and yellow colors were used to demonstrate discreet perfusion through flangeless nuts 1/4-28 (red connectors), while plugs (black connectors) were used to block flow in specific ports.

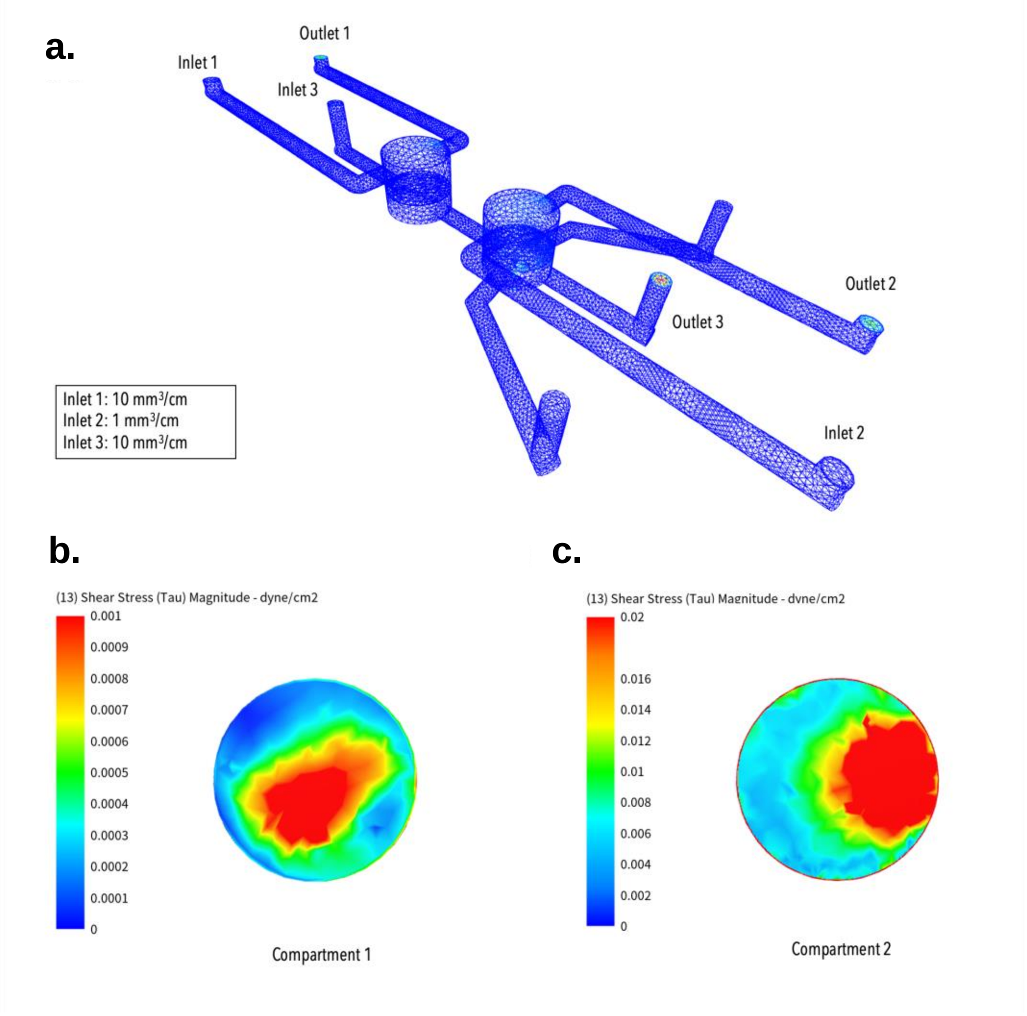

Fig. S.4. **Shear stress simulation on the membrane surface a.** Overview of the microfluidic device depicting its fluidic connections as well as the control volumes generated with the automatic mesh generator. **b.** Shear stress distribution at the surface of the integrated PET membrane in compartment 1. **c.** Shear stress distribution at the surface of the integrated PET membrane in compartment 2.

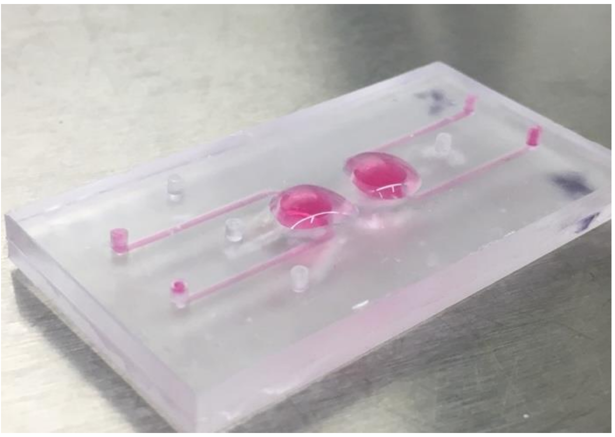

Fig. S.5. 3D-printed unit used to evaluate cell biocompatibility and toxicity. In order to simplify the validation of the effect of the 3D-printing resin on cells, we 3D-printed the upper unit of the MOC without the nuts and cover. A porous membrane was placed between the top and bottom units which then were bonded together.

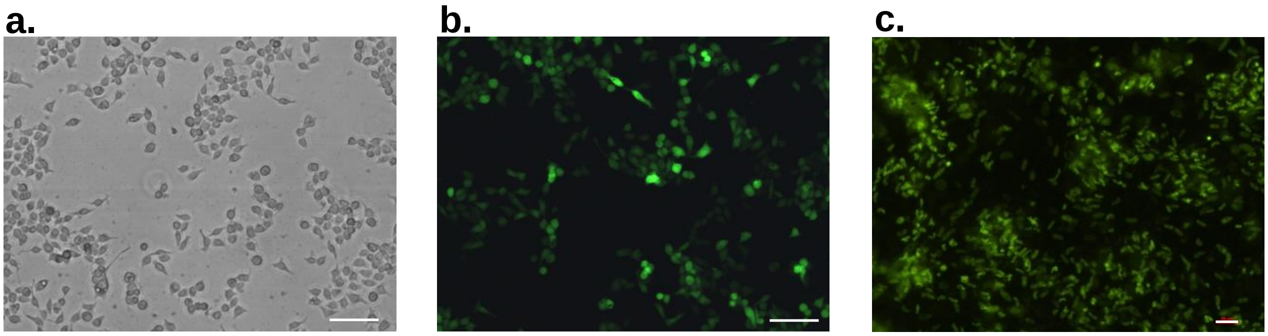

Fig. S.6. Fluoresce Visualization of viable GFP-PC9 cells. a. Bright field and GFP visualization of viable GFP-PC9 cells **b.** cultured on plate and **c.** when introduced into the device and cultured on PET membrane with standard fluoresce microscopy. scale bar = 50 µm
